# Treatment of hepatocellular carcinoma with gluteus medius metastasis: a case report and a literature review

**DOI:** 10.3389/fonc.2025.1329756

**Published:** 2025-05-01

**Authors:** Yanxin Li, Ji Luo, Jianyong Zhang, Mingqiang Sun, Jinping Yang

**Affiliations:** ^1^ Department of Oncology, First People’s Hospital of Guangyuan, Guangyuan, China; ^2^ Department of Obstetrics and Gynecology, The First People’s Hospital of Guangyuan, Guangyuan, China

**Keywords:** hepatocellular carcinoma, muscle metastasis, radiotherapy, interstitial brachytherapy, bulky tumor

## Abstract

Hepatocellular carcinoma (HCC) accounts for 85%–90% of all primary liver cancers (PLCs). Owing to the occult nature of HCC, most patients present at an advanced stage at the time of initial diagnosis and have a poor prognosis. With regard to systemic therapy, targeted therapy and immunotherapy are currently the centers of clinical research. With regard to local treatment, surgical resection, radiofrequency ablation, hepatic artery chemoembolization, and radiotherapy are commonly used. Interstitial brachytherapy is commonly used for the treatment of cervical and genitourinary cancers. In this case, interstitial brachytherapy was used to treat gluteus medius muscle metastasis from PLC, with good local control and symptom relief.

## Introduction

Interstitial brachytherapy is a treatment method in which a hollow metal needle is directly inserted into the tumor tissue and connected to a radiation source through the connecting tube. It allows tumor tissue to receive a high dose of radiation. Interstitial brachytherapy is a common form of cervical cancer treatment that can be used for the local treatment of other superficial tumors.

## Case report

**Figure 1:**
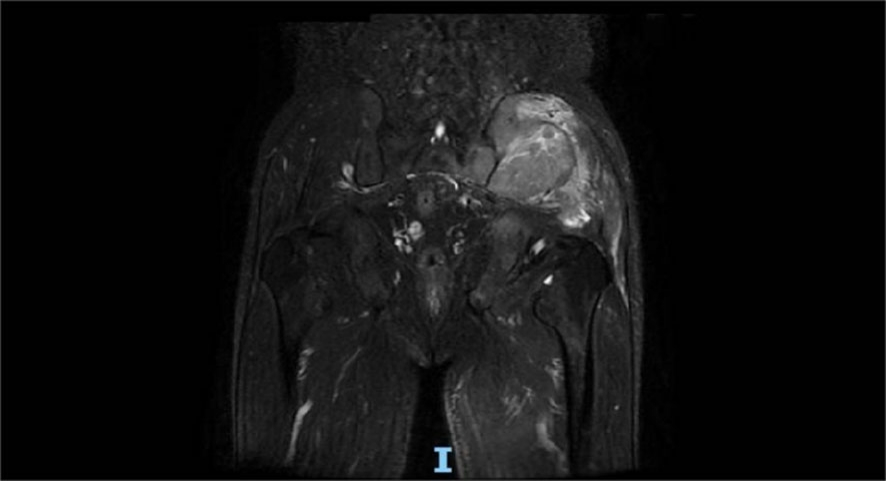
Pelvic MRI (11 July 2022) revealed a malignant mass approximately 9.2 cm × 4.1 cm in the left gluteus medius region.

**Figure 2:**
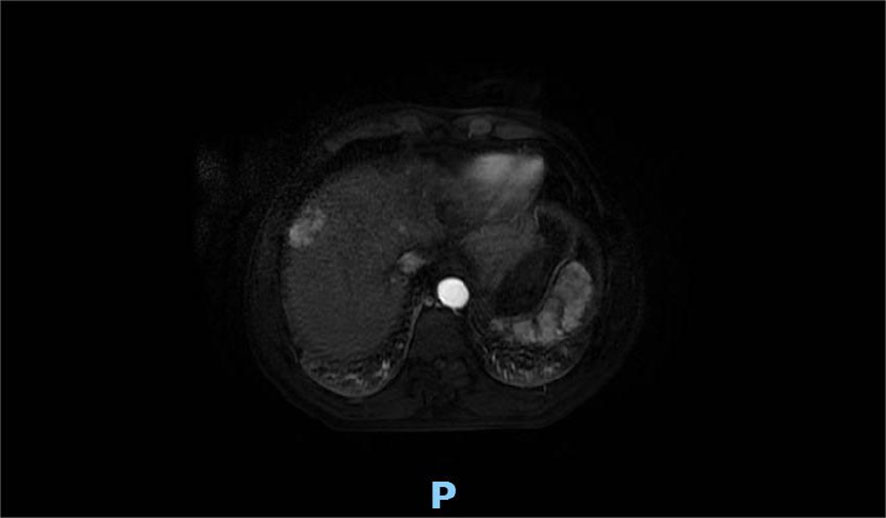
Upper abdominal MRI (20 July 2022) showed the liver occupancy in S7 and S8 segments (approximately 3.6 cm × 2.2 cm, 3.1 cm × 2.3 cm) probably refer to HCC.

**Figure 3:**
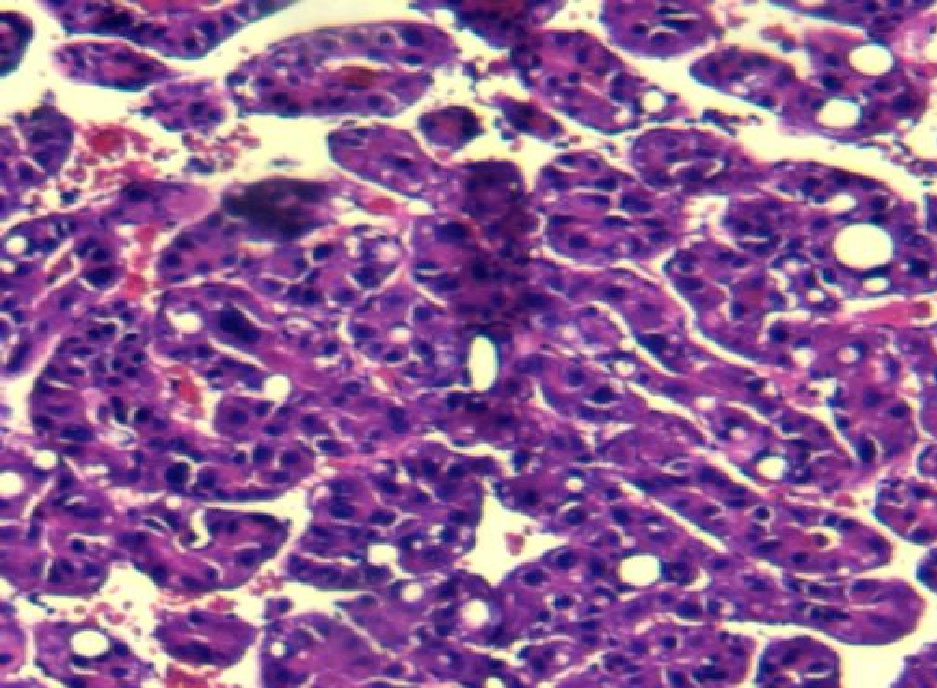
Pathological results of the left gluteal mass: metastatic and moderately differentiated hepatocellular carcinoma. The immunohistochemistry results of tumor were: CK7 (−), CK20 (−), CEA (−), CD10 (bile duct +), CD34 (sinusoidal endothelial +), hepatocyte (+), Glypican-3 (+), Syn (−), CGA (−), CK8/18 (−), P53 (wild type), kI67 (10% +).

**Figure 4:**
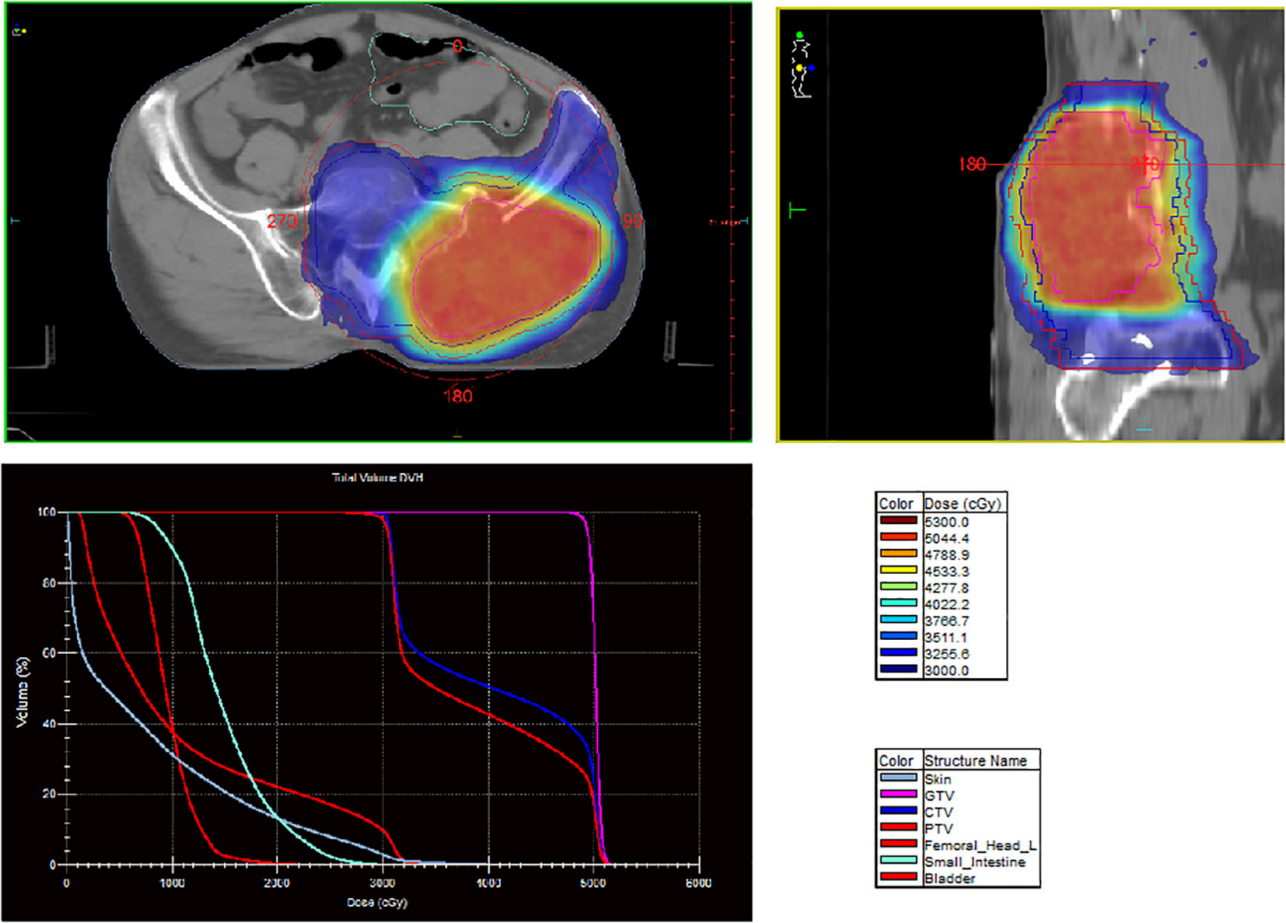
Three-dimensional dose distribution and DVH of the left buttock.

A 60-year-old man presented with persistent pain in the left buttock for the past month. Pelvic MRI (11 July 2022) revealed a malignant mass approximately 9.2 cm × 4.1 cm in the left gluteus medius region (**Figure 1**). The mass was poorly demarcated from the piriformis muscle, and multiple soft tissue signals were observed in the left ilium and sacrum. The left sacroiliac joint space is visible. Upper abdominal MRI (20 July 2022) showed a tumor in liver segments S7 and S8 (approximately 3.6 cm × 2.2 cm, 3.1 cm × 2.3 cm) concerning for HCC (**Figure 2**). The patient had a history of chronic hepatitis B for 10 years and had been treated with oral entecavir for a long time. However, the patient did not visit the hospital for regular examinations. The patient had neither been previously diagnosed with HCC nor received any treatment for the condition. Before performing a biopsy, we also considered osteosarcoma, which typically presents as a localized painful mass arising from skeletal muscle but tends to occur in adolescents. HCC was diagnosed using CT-guided fine-needle aspiration biopsy. Pathological analysis of the left gluteal mass by immunohistochemistry demonstrated metastatic, moderately differentiated HCC. The immunohistochemistry results for tumors were as follows: CK7 (−), CK20 (−), CEA (−), CD10 (bile duct +), CD34 (sinusoidal endothelial +), hepatocyte (+), Glypican-3 (+), Syn (−), CGA (−), CK8/18 (−), P53 (wild type), ki67 (10% +) (**Figure 3**). Head and chest CT, abdominal color Doppler ultrasound, and lumbosacral MRI were performed. In addition to metastasis of the gluteus medius, lumbar level 3 vertebral body metastasis was observed. The patient was diagnosed as having HCC with metastasis to the left gluteus medius. The patient’s pain was persistent, localized to the left buttock and left lower extremity, and accompanied by numbness. Given the patient’s pain in the left gluteus and the size of the gluteal metastasis with obvious compression symptoms and few surrounding organs at risk, a high dose of external beam radiotherapy was chosen (PTV 48 Gy/12f) to the left gluteus starting on 19 July 2022 (**Figure 4**). Palliative radiotherapy was not considered in the lumbar 3 vertebral body because vertebral metastasis was not painful.

**Figure 5:**
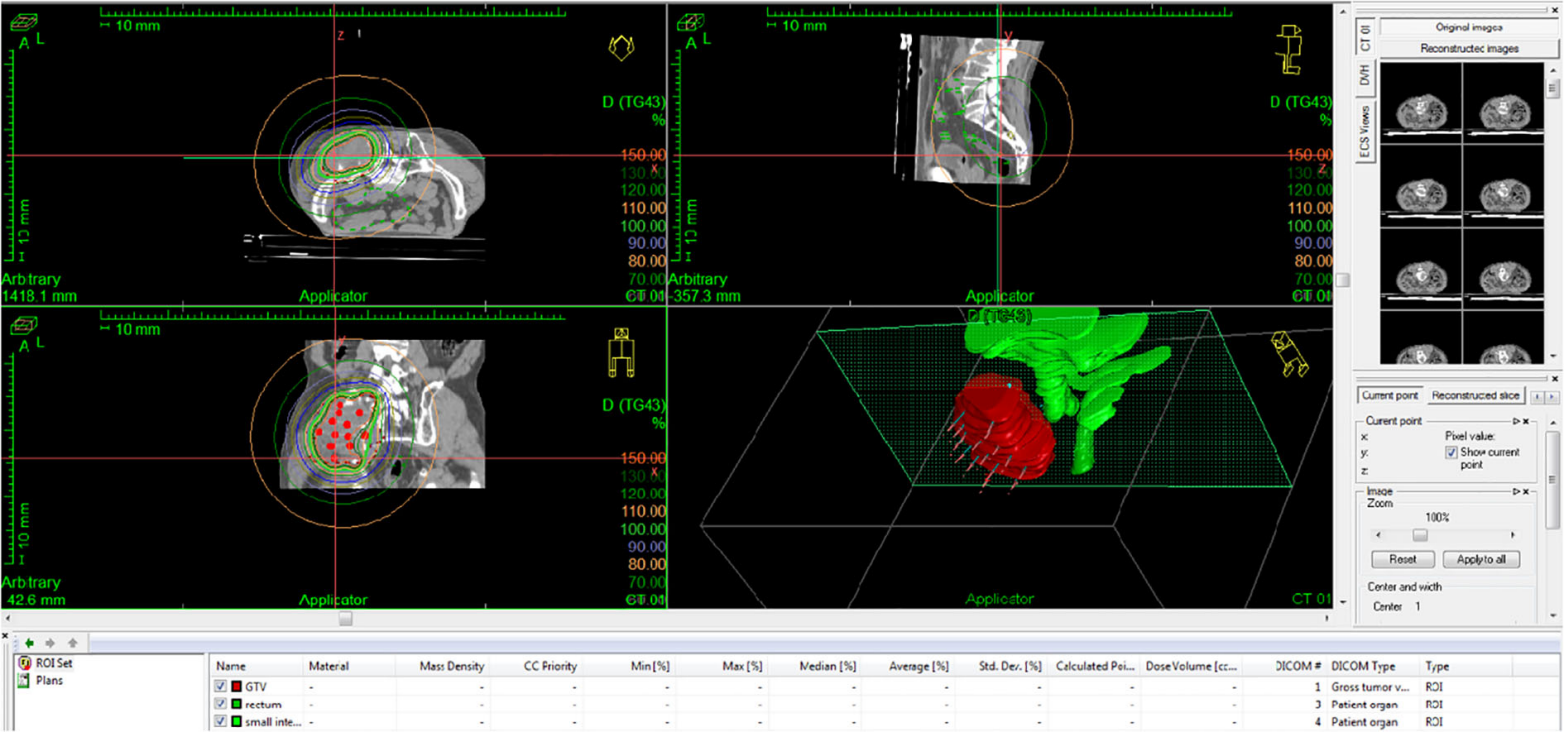
Screenshot of the left buttock brachytherapy plan.

At the same time, 8 mg of lenvatinib mesilate capsules were administered orally for 1+ months (from 19 July 2022 to 13 August 2022), and 200 mg of sintilimab was administered intravenously on 1 September 2022, to the patient. The pain and numbness improved after EBRT but could not be completely relieved. As the gluteal tumor remained substantial after completion of EBRT, the patient was scheduled to return for additional treatment with interstitial brachytherapy. On 5 September 2022, interstitial brachytherapy of the left buttock lesion was performed (16 Gy/1f) (**Figure 5**). After combined spinal and epidural anesthesia, 16 needles were placed in the patient’s left buttock. The D2cc dose of the small intestine and rectum was limited to 7 Gy, and the cobalt-60 radioactive source was used to deliver the target dose of D90 16 Gy, which was approximately 20 min.

**Figure 6:**
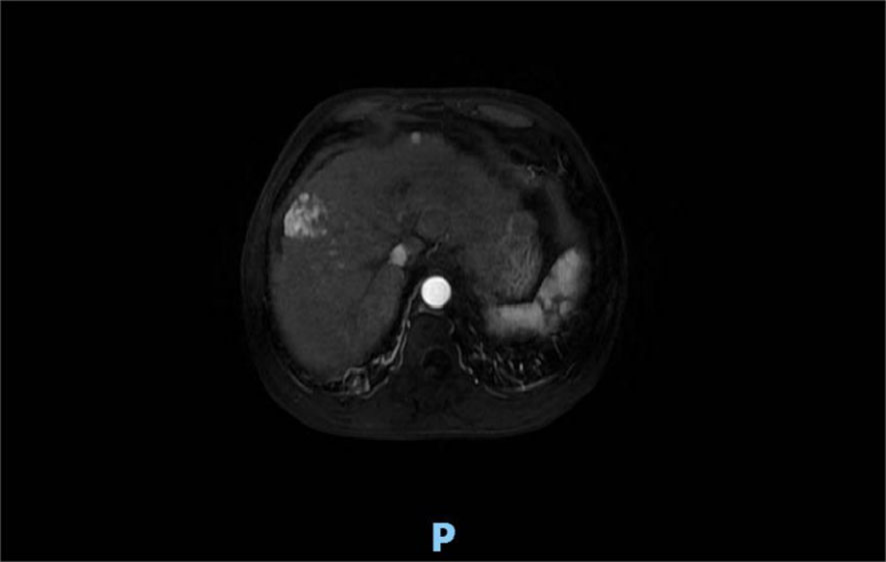
Four months after treatment, hepatic MRI (16 January 2023) showed no significant change compared with the previous (20 July 2022).

**Figure 7:**
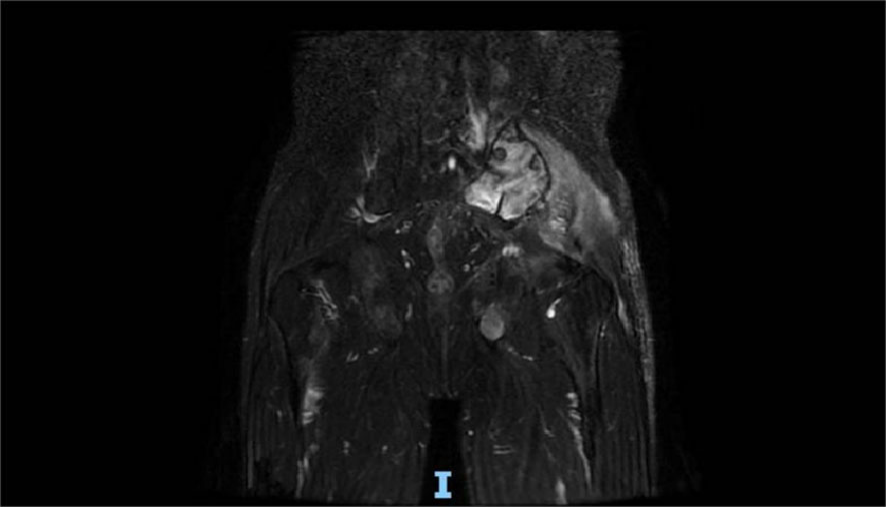
Pelvic MRI (16 January 2023) showed that the volume of lesions was smaller than that before (11 July 2022) in the left buttock after treatment, which is approximately 6.5 cm × 2.3 cm.

Four months after treatment, hepatic MRI (16 January 2023) (**Figure 6**) showed no significant change compared with the previous MRI (20 July 2022). Pelvic MRI (16 January 2023) (**Figure 7**) showed that the volume of left buttock lesion was smaller after treatment compared to prior (11 July 2022), now measuring approximately 6.5 cm × 2.3 cm, a significant reduction from the initial size of 9.2 cm × 4.1 cm.

After combined radiotherapy with EBRT and interstitial brachytherapy, although systemic therapy was interrupted for personal reasons (only one cycle of systemic therapy was administered and no further treatment was given thereafter), the tumor in the left gluteus medius region remained significantly reduced, even after 4 months (**Table 1**).

**Table 1:** Charts showing timelines of relevant data during treatment.

Time	2022-07-11	2022-07-20	2022-07-19to2022-8-13	2022-09-01	2022-09-05	2023-01-16	2023-01-16
Therapeutic measures			Radiotherapy for the left buttock and Lenvatinib mesilate capsules was administered orally	Sintilimab was administered intravenously	Left buttock Interstitial brachytherapy		
Size of the mass in MRI	Mass in left hip: 9.2cm×4.1cm	Mass in liver: 3.6cm×2.2cm in S7, 3.1cm×2.3cm in S8				Mass in left hip: 6.5cm×2.3cm	Mass in liver: 3.7cm×2.1cm in S7, 3.1cm×2.3cm in S8

## Discussion

PLC is one of the most common malignancies in the world. The annual number of new cases of PLC worldwide is 841,000, ranking 6th in incidence and 3rd in cause of death. It is believed to be related to liver cirrhosis, viral hepatitis, aflatoxins, and other chemical carcinogens (1). Approximately 90% of PLC cases are HCC (2). More than 2/3 of HCC patients who present with advanced disease are unable to receive radical treatment such as surgical resection (3). Instead, these patients may receive transcatheter arterial chemoembolization, local radiotherapy, radiofrequency ablation, targeted therapy, or immunotherapy.

Currently, surgery plays a key role in the treatment of HCC. Surgery is the first-line therapy for HCC with a diameter ≤5 cm. However, due to frequent late presentation of the disease, portal hypertension, and poor liver function among other reasons, less than 30% of patients can undergo hepatectomy (4). Radiofrequency ablation is less invasive and can be repeated, and local ablation can be used as first-line therapy for hepatocellular carcinoma located within the liver ≤3 cm or two to three lesions (5).

Transcatheter arterial chemoembolization (TACE) is an important treatment modality for HCC. However, because of the dual blood supply to the liver, TACE has a good effect on tumors supplied by the hepatic artery but may have a poor effect on tumors supplied by the portal vein. TACE combined with local radiotherapy can effectively improve the local control rate and survival of patients with advanced HCC (6, 7).

Earlier forms of external beam radiotherapy often result in radiation-induced liver cell damage with a poor curative effect compared to more focal radiation modalities. Radiation-induced liver disease (RILD) has been shown to be associated with whole-liver irradiation doses greater than 30 Gy (8). The lethal dose for liver tumors is approximately 60 Gy/30f (9). The dose of conventional radiotherapy for HCC is 50 Gy–75 Gy (28). With the improvement of radiotherapy equipment and optimization of the target dose allowing for greater precision, more ablative doses to the tumor may be achieved while protecting the normal liver tissue. This allows for an improvement in local control (10). Radiotherapy is actively used in the treatment of HCC, with studies showing that the local control rate of HCC with radiotherapy at 1 year or 2 years is close to 90% with ablative doses, i.e., stereotactic body radiation therapy (SBRT) (10).

SBRT is a noninvasive treatment modality with a wide range of indications. Furthermore, studies have shown that the rate of local control of SBRT is comparable to that of radiofrequency ablation in HCC (11–13). Neoadjuvant radiotherapy has been shown to significantly reduce the recurrence and mortality of HCC compared with surgery alone (14). Intraoperative radiation therapy (IORT) is a technique in which a single large dose of radiation is administered intraoperatively directly to the tumor tissue and suspected tumor areas following tumor resection to maximize the killing of residual tumor cells. This radiotherapy method can precisely locate and increase the dose to the target area, protect normal tissue, and reduce the complications of radiotherapy. IORT can reduce the risk of postoperative recurrence by eliminating residual disease during surgery,. The biological effect of the same dose of intraoperative radiotherapy is 2.5 times greater than that of conventional external-beam radiotherapy (15).

Residual tumors and local recurrence after surgical treatment remain major challenges in the treatment of HCC (16). Adjuvant radiotherapy after hepatectomy may lead to better survival in patients with HCC (17, 18, 29).

Targeted therapeutic agents may prolong the survival of patients with unresectable hepatocarcinomas (19, 20); however, there are limited data on radiotherapy combined with targeted therapy.

Brachytherapy is a form of radiation therapy in which radioactive sources are placed in or near the treatment site. Commonly used radioactive sources include cobalt-60, iridium-192. Brachytherapy is widely used for the treatment of cervical, prostate, and breast cancers. The greatest feature of brachytherapy is that it affects only a very limited area around the radioactive source. Therefore, the radiation dose to normal tissues that are distant from the radioactive source can be reduced. Depending on the placement method of the radioactive source, it can be divided into interstitial implantation and contact types. In interstitial brachytherapy, the radioactive source is placed directly into the target tissue, similar to prostate cancer. In contact brachytherapy, a radioactive source is placed in a space close to the target tissue. This space can be a cavity in the body such as the vagina.

Radioactive seed implantation is another local treatment for HCC, which includes inter-tissue implantation, portal vein implantation, inferior vena cava implantation, and biliary tract implantation. Compared to conventional external irradiation, radioactive particle implantation has the advantages of a high local dose to the tumor and less radiation exposure to the normal surrounding tissue.

HCC is a highly aggressive tumor, and extrahepatic metastasis is not rare. Common sites include the lungs, brain, lymph nodes, bones, and adrenal glands (21, 30). The incidence of skeletal muscle metastasis of HCC is low. HCC usually metastasizes through direct invasion or hematogenous spread via intrahepatic vessels or lymphatic channels. At present, there are only a few case reports on skeletal muscle metastasis from HCC (22–27) (**Table 2**), most of which were treated with surgery to relieve local symptoms. Radiation was used in only a few cases. Jiang et al. (23) reported a case of HCC with ocular muscle metastasis. Three months before the visit, the patient developed progressive exophthalmos and diplopia, and adduction and depression in both eyes were severely limited. Orbital MRI revealed bilateral multiple nodular enlargements of the external oblique muscles. A biopsy of the right superior oblique muscle suggested HCC metastasis. The patient was treated with orbital external beam radiation therapy, and bilateral proptosis and ocular motility significantly improved. External beam radiation therapy can improve ocular symptoms and control the development of the disease. Takahashi et al. (26) reported a case of HCC with left paravertebral muscle metastasis. The patient developed back pain after receiving sorafenib for HCC. A CT scan revealed a 3.7 cm enhancing lesion in the left paravertebral muscle. Biopsy of the mass revealed metastatic HCC. The patient was treated with stereotactic hypofractionated image-guided radiation therapy (SHFRT) (40 Gy/4f/10 Gy). The paravertebral muscle metastasis was stable 3 months after radiotherapy. The patient achieved good local control after radiotherapy, which avoids the risk of paraplegia that may occur during surgical treatment. Jo et al. (27) reported a case of HCC with multiple muscle metastases, including the left pectoralis major muscle, deltoid muscle, left teres minor muscle, and right deltoid muscle. The patient received seven courses of external radiotherapy for subcutaneous tissue, muscle, and bone metastases and systemic therapy with sorafenib over a period of three years, and achieved good results. In this case, the patient was able to undergo repeated radiation therapy, which was almost impossible during surgical treatment. Surgical treatment requires the patient to have an excellent physical condition, better economic condition, and the ability to avoid various surgical risks. However, radiotherapy enables patients to receive repeated local treatments in a relatively short period of time.

**Table 2:** Case reports of skeletal muscle metastasis from HCC.

Reference	Year	Locus of muscular metastasis	Treatment for metastasis
(23)	2012	Ocular muscle	Orbital external beam radiation therapy
(26)	2017	Left paravertebral muscle	Stereotactic hypo-fractionated image-guided radiation therapy
(27)	2013	Left pectoralis major muscle, deltoid muscle, left teres minor muscle, and right deltoid muscle	Multiple external radiotherapy and systemic treatment with sorafenib
(22)	2011	Right humerus muscle	Surgical resection
(24)	2013	Right hypochondrium	Palliative chemotherapy
(25)	2019	Biceps femoris muscle	Surgical resection

This case involved a patient with advanced liver cancer who presented with distant metastases to the vertebral body and skeletal muscle. Therefore, systemic therapy is the primary treatment option. Because of obvious local symptoms in the left buttock, local treatment was administered. Most previously reported cases of HCC skeletal muscle metastasis were treated with surgery to improve local symptoms, and only a few cases were treated with radiotherapy. Compared with surgical treatment, local radiotherapy is associated with less trauma, faster recovery, and is more acceptable to patients, which is also a common treatment method.

For large metastatic lesions, external beam radiotherapy alone has a limited therapeutic effect, while palliative interstitial brachytherapy combined with external beam radiotherapy can control local lesions and improve clinical symptoms. In this case, external radiotherapy combined with local interstitial brachytherapy was used to treat metastatic HCC in the gluteus medius muscle region. The local tumor lesions were controlled and the patient’s symptoms were significantly relieved. This case demonstrates that interstitial brachytherapy may significantly improve local tumor control and result in greater symptom relief in the palliative setting. This provides new ideas for palliative treatment of local tumors in the clinic. For giant muscle metastases, surgery carries the risks of large trauma, slow recovery, and difficult healing of postoperative cancer wounds. Local radiotherapy provides clinicians with an alternative treatment approach. Interstitial brachytherapy can further shrink the lesion, improve the local tumor control rate, and achieve better clinical treatment effects without significantly affecting surrounding normal organs. Therefore, for large muscle metastases from advanced malignant tumors, external beam radiotherapy combined with interstitial brachytherapy can be used to control local lesions and improve clinical symptoms.

## Conclusions

Advances in radiation technique and technology have improved the efficacy and safety of radiotherapy for PLC. The efficacy and safety of radiotherapy for PLC is increasing. Radiotherapy alone or in combination with other therapies has greatly improved the local control of HCC and survival rate of patients, and the value of radiotherapy in the treatment of HCC has been increasingly recognized (10, 14). For superficial skeletal muscle metastases from HCC, interstitial brachytherapy may improve local tumor control and provide greater relief of symptoms, which provides a new idea for our clinical treatment.

## Data Availability

The original contributions presented in the study are included in the article/supplementary material. Further inquiries can be directed to the corresponding author.
